# Retrospecting Digital Media Use, Negative Emotions, and Trust Gaps During the COVID-19 Pandemic in China: Cross-Sectional Web-Based Survey

**DOI:** 10.2196/49422

**Published:** 2024-07-10

**Authors:** Lu Wei, Qing Huang

**Affiliations:** 1 School of Journalism and Communication Communication University of Zhejiang Hangzhou China; 2 International Communication Institute College of Media and International Culture Zhejiang University Hangzhou China

**Keywords:** digital media use, negative emotions, family members–strangers trust gap, family members–acquaintances trust gap, mediation effect, COVID-19

## Abstract

**Background:**

Retrospecting the trust gaps and their dynamics during the pandemic is crucial for understanding the root causes of postpandemic challenges and offers valuable insights into preparing for future public health emergencies. The COVID-19 pandemic eroded people’s trust in strangers and acquaintances, while their trust in family members remained relatively stable. This resulted in 2 trust gaps, namely, the family members–strangers trust gap and the family members–acquaintances trust gap. Widening trust gaps impede social integration and undermine the effective management of public health crises. However, little is known about how digital media use shaped trust gaps during a pandemic.

**Objective:**

This study aims to investigate the relationships between digital media use, negative emotions, the family members–strangers trust gap, and the family members–acquaintances trust gap during the COVID-19 pandemic in China. We test the mediating role of negative emotions between digital media use and 2 trust gaps and compare the indirect effect of digital media use on 2 trust gaps through negative emotions.

**Methods:**

A cross-sectional web-based survey was conducted in China between January 31, 2020, and February 9, 2020. A total of 1568 adults participated in the survey. Questions related to digital media use, negative emotions, trust in family members, trust in acquaintances, and trust in strangers during the pandemic were asked. Regression analyses were performed to test the associations between the examined variables. We used a 95% bootstrap CI approach to estimate the mediation effects.

**Results:**

Digital media use was positively associated with negative emotions (*B*=0.17, SE 0.03; *P*<.001), which in turn were positively associated with the family members–strangers trust gap (*B*=0.15, SE 0.03; *P*<.001). Likewise, digital media use was positively associated with negative emotions (*B*=0.17, SE 0.03; *P*<.001), while negative emotions were positively associated with the family members–acquaintances trust gap (*B*=0.08, SE 0.03; *P*=.01). Moreover, the indirect effect of digital media use on the family members–strangers trust gap (*B*=0.03, SE 0.01; 95% CI 0.01-0.04) was stronger than that on the family members–acquaintances trust gap (*B*=0.01, SE 0.01; 95% CI 0.003-0.027).

**Conclusions:**

The results demonstrate that negative emotions resulting from the frequent use of digital media are a key factor that accounts for the widening trust gaps. Considering the increasing reliance on digital media, the findings indicate that the appropriate use of digital media can prevent the overamplification of negative emotions and curb the enlargement of trust gaps. This may help restore social trust and prepare for future public health crises in the postpandemic era.

## Introduction

### Background

During the period from early 2020 to May 5, 2023, marked by the World Health Organization’s declaration of the end of the COVID-19 global health emergency [1], the pandemic caused increased societal division and declined social trust across many countries [2]. The erosion of social trust during a crisis tends to engender enduring repercussions, such as individuals’ psychological distress, public dissent, and a decrease in community resilience [3,4]. This, in turn, impedes the processes of social cohesion and the maintenance of a robust society. Moreover, against the backdrop of a high-risk environment [5], large-scale infectious diseases might break out in the future. Therefore, retrospecting social trust dynamics and examining the influence of various factors on trust-related outcomes during the pandemic have dual implications: (1) to understand the root causes of postpandemic trust-related issues; and (2) to prepare for future public health emergencies, given that trust serves as the bedrock of effective crisis management [6,7].

We used the individual-level approach to understand social trust [8-10] and define it as an individual’s trust in others, including family members, acquaintances (eg, coworkers, classmates, and casual friends), and strangers. Noticeably, the differential mode of association (*chaxugeju*) in Chinese culture indicates that as social distance between individuals increases, trust decreases [11,12]. Accordingly, trust is highest and most stable in family members, followed by acquaintances, while trust in strangers is relatively low and more susceptible to change. This phenomenon suggests 2 trust gaps: the family members–strangers trust gap and the family members–acquaintances trust gap. Moreover, evidence suggests that these gaps existed during the COVID-19 pandemic [13,14]. Given that increasing trust gaps are linked to social estrangement and other challenges [15], it is essential to examine how the trust gaps widened during the pandemic.

Information that circulates on digital media platforms is a major source that influences trust gaps. At the early stage of the pandemic, information on digital media involved a considerable amount of uncivil comments about general others [16-18]. Thus, an individual’s exposure to such information might erode his or her trust in strangers and in acquaintances, whereas their trust in family members was less likely to be affected. This enlarged the family members–strangers trust gap and the family members–acquaintances trust gap. Besides, an individual’s exposure to information on digital media that highlighted the susceptibility and severity of the coronavirus and the social consequences of the pandemic provoked his or her negative emotions, such as fear, anxiety, sadness, anger, and hostility [19,20], which in turn weakened their trust in others [21,22], including trust in strangers and acquaintances. Therefore, digital media use is a major informational source that influences the 2 trust gaps, while negative emotions are a psychological response that may increase these gaps.

This study aims to unravel the relationship between digital media use, negative emotions, the family members–strangers trust gap, and the family members–acquaintances trust gap during the pandemic. Despite the extensive body of studies that examined media use and its associated outcomes during the COVID-19 pandemic [23-25], this study is, to our knowledge, the first to propose the concept of *trust gap* and explore the mechanisms that contribute to the increasing gaps. Moreover, our study is an empirically grounded theoretical exploration of the trust gaps and its associated dynamics, rather than policy research. Therefore, although the pandemic has ended, our findings and conclusions may advance the understanding of the effect of media use on trust-related challenges in other health and risk situations beyond the COVID-19 pandemic.

### Negative Emotions Mediate Between Digital Media Use and 2 Trust Gaps

Digital media refer to a wide range of computer-mediated and mobile technology–based applications. These encompass websites, search engines, web-based forums, wikis, blogs, social networking sites, news feeds, video-sharing sites, and so forth [26,27]. People use digital media to satisfy certain needs, such as cognition, entertainment, interactivity, and agency [28-30]. Among various purposes of digital media use, information seeking serves as the basic motive to meet people’s cognitive need [31-33]. In particular, acquiring mediated information and staying informed of the latest updates is of great significance during a public health emergency [34,35]. Drawing upon previous conceptions of media use for information seeking [36,37], we define digital media use as the frequency that people use different digital media tools to acquire information related to the coronavirus. This information mainly involves the health risks of the virus and the socioeconomic consequences of the pandemic.

Despite the existence of positive emotions, people tend to feel negative emotions more commonly when a contagious disease breaks out. For instance, people experienced worry, anxiety, fear, distress, anger, and sadness at the early stage of the Middle East respiratory syndrome (MERS), and H1N1 influenza and H7N9 influenza [35,38,39]. Given that the coronavirus is characterized by high infection rate and severe consequences, people tended to feel fear, anxiety, and sadness during early phase of the pandemic. Moreover, the unsettled disputes over the origin of the pandemic and the widespread use of controversial, xenophobia, and stigmatized terms associated with the coronavirus incited people’s anger and hostility [16-18]. Thus, we define negative emotions as people’s undesirable feelings toward the coronavirus outbreak and its consequences, such as fear, anxiety, sadness, anger, and hostility.

A considerable amount of literature has discussed the association between people’s digital media use and their negative emotions amid public health emergencies. For example, studies demonstrated that the more frequently individuals exposed themselves to social media information, the stronger their negative emotions were, either in the MERS outbreak in South Korea [38] or during the H7N9 influenza in China [39]. Moreover, research demonstrated that Chinese netizens’ frequent digital media use significantly amplified their negative emotions at the early stage of the COVID-19 outbreak, especially their anxiety, depression, and stress [19,20]. When the coronavirus first broke out in China, information about increasing number of confirmed cases, threat to public health, disruption to daily routines, and economic downturn circulated widely across a variety of digital media [40]. Through exposure to information about the coronavirus’s unpredictable health threat, people felt anxiety, fear, and sadness. Similarly, consuming information that emphasized the socioeconomic consequences of the pandemic was likely to trigger people’s anger and hostility toward those who were attributed with the responsibility of accelerating the spread of the virus. Accordingly, the more frequently people used digital media to acquire information, the stronger their negative emotions were.

When individuals are exposed to a crisis, their negative emotions motivate them to attribute the causality to certain actors [22]. In circumstances in which the cause of an event remains unclear, an individual’s attribution-dependent negative emotions are more likely to encourage him or her to attribute the responsibility to socially distant ones [41]. Given that the disputes over the origin and cause of the coronavirus have remained unsettled so far [42], the pandemic can be seen as a health crisis without an explicit or agreed-upon cause. Thus, people’s negative emotions about the pandemic tend to motivate them to attribute part of the responsibility to socially distant others. According to the attribution theory, people who are attributed to responsibility are considered distrustful [21]. Moreover, evidence showed that negative emotions provoked by contagious diseases led people to be distrustful of out-group members [43-45]. Because strangers and acquaintances were regarded as socially distant others or out-group members during the pandemic, the negative emotions tended to decrease one’s trust in them, thereby enlarging the family members–strangers trust gap and the family members–acquaintances trust gap.

Taken together, the literature reviewed in this section indicates the mediating role of negative emotions between digital media use and the 2 trust gaps. Thus, we posit the following hypotheses:

Hypothesis 1: negative emotions mediate the association between digital media use and the family members–strangers trust gap.

Hypothesis 2: negative emotions mediate the association between digital media use and the family members–acquaintances trust gap.

**Figure 1:**
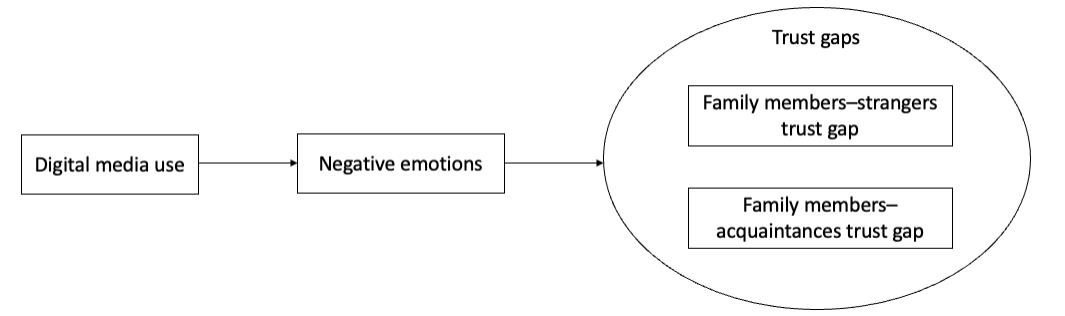
The conceptual model.

Moreover, because strangers were viewed as more socially distant others than acquaintances [12], it was likely that individuals attributed more responsibility to strangers than to acquaintances when experiencing negative emotions elicited by digital media use, thus leading to varying degrees of increase in the 2 trust gaps. Hence, we put forward the following hypothesis:

Hypothesis 3: the mediation effect of negative emotions between digital media use and the family members–strangers trust gap is stronger than the mediation effect of negative emotions between digital media use and the family members–acquaintances trust gap. Figure 1 depicts the conceptual model.

## Methods

### Participants and Procedures

A cross-sectional web-based survey was conducted from January 31, 2020, to February 9, 2020, an early phase of the COVID-19 outbreak in China. We chose this time span for several reasons. First, a considerable amount of information related to COVID-19 was circulating on a variety of digital media platforms during this period of time, which guaranteed that respondents had adequate informational resources to consume. Second, given the important role of negative emotions in the conceptual model, we attempted to capture people’s negative emotions at an early stage to avoid the emotional burnout that might occur at a later stage of the pandemic. Third, public understanding of the coronavirus was very limited at the very beginning of the outbreak. Insufficient knowledge led the public to experience a high degree of uncertainty and a strong sense of insecurity, which potentially impacted their social trust [46,47]. This warranted the investigation into the relationship between digital media use and trust gaps at the very early phase compared with other phases of the health crisis.

We commissioned Sojump, a professional web-based survey company in China, to collect survey data. The company provides a sampling service of 6.2 million registered respondents distributed throughout China. We used a random sampling strategy within the survey pool. Given the emergency nature of the COVID-19 outbreak, this sampling strategy was an efficient and timely manner to explore initial public reactions and attitudes toward the pandemic. Moreover, previous studies have used this sampling strategy to examine major health risk issues in China [48-50].

In terms of the sampling procedure, Sojump first randomly selected 2840 users from the 6.2 million-user survey pool and asked them to participate in the web-based survey through an email invitation. A total of 1656 respondents finished the questionnaire, with a response rate of 58.3% (1656/2840). After deleting the cases with missing values or those that did not pass the attention checks, we obtained a sample of 1568 valid cases for data analysis.

The sample covered 31 provinces, municipalities, and autonomous regions across the Chinese mainland. Because we focused on people’s digital media use and the associated effects, Chinese netizens were regarded as the population [51]. Our sample was representative of the population on gender (779/1568, 49.7% were females) and fairly representative on age (739/1568, 47.1% were younger than 30 years) and income (579/1568, 37.0% with an income of 3000-8000 Chinese Yuan (CNY; US $412.60-$1100.28). However, because college students and graduates constituted a major part of the survey pool, we oversampled those with greater education attainment (1237/1568, 78.9% were college students or graduates or above). Table 1 compares the demographic features of our sample with those of the population.

**Table 1:** Sample demographics compared with the demographic features of Chinese netizens^a^.

Variables		Sample, n (%)	Proportion of the population, %
Gender (female)		779 (49.7)	48.1
**Age (years)**			
	<30	739 (47.1)	44.7
	30-49	754 (48.1)	38.4
	>50	75 (4.8)	16.9
**Income (CNY^b^)**			
	≤1000	128 (8.2)	27.9
	1001-3000	644 (41.0)	23.1
	3,001-5000	332 (21.2)	21.5
	5001-8000	247 (15.8)	14.3
	>8000	217 (13.9)	13.3
**Education**			
	Middle school or below	54 (3.4)	58.3
	High school/technical school	277 (17.7)	22.2
	College or above	1237 (78.9)	19.5

^a^The demographic features of Chinese netizens were retrieved from the 45th Statistical Report on China’s Internet Development issued in April 2020.

^b^CNY: Chinese Yuan; 1 CNY=US $0.14.

### Ethical Considerations

The institutional review board of Zhejiang University approved the data collection protocol (2020-056). Voluntary informed consent was obtained from the participants before the web-based survey. Each participant was compensated with CNY 12 (US $1.6) for participating in the study. The final data set was anonymized. We ensured that no identifiable private information was linked to the participants.

### Measures

#### Trust Gaps

Trust gaps included the family members–strangers trust gap and the family members–acquaintances trust gap. First, we separately measured respondents’ trust in their family members, acquaintances, and strangers. Drawing upon the extant measurement [52], trust in family members (mean 4.53, SD 0.71), trust in acquaintances (mean 3.85, SD 0.75), and trust in strangers (mean 2.35, SD 0.81) were measured with a single item asking respondents the degree to which they trusted their family members, acquaintances, and strangers during the COVID-19 outbreak, respectively. A 5-point Likert scale that ranged from 1 (totally distrust) to 5 (totally trust) was used. Then, the family members–strangers trust gap was measured by subtracting trust in strangers from trust in family members (mean 2.18, SD 1.02), and the family members–acquaintances trust gap was measured by subtracting trust in acquaintances from trust in family members (mean 0.68, SD 0.93).

#### Digital Media Use

Drawing upon the measurement of digital media use for informational purpose [37], we measured digital media use as the frequency of people’s exposure to COVID-19 information through a wide range of digital media tools. Based on people’s habits of digital media use during the pandemic [40], we asked respondents how frequently they exposed themselves to information about COVID-19 via the nine digital media tools on a 5-point Likert scale ranging from 1 (never) to 5 (very frequently): (1) portal websites, (2) social networking sites, (3) news apps, (4) video apps, (5) web-based question-and-answer communities, (6) search engines, (7) government news apps, (8) medical apps, and (9) netizens-run digital media. Nine items were averaged to form a composite index of digital media use (mean 3.35, SD 0.63; Cronbach α=0.70).

#### Negative Emotions

According to the commonly experienced negative emotions during public health emergencies [38,53], this study measured negative emotions by asking respondents to indicate the degree to which they felt, on a 5-point Likert scale ranging from 1 (not at all) to 5 (very strongly), the following five emotions toward the COVID-19 outbreak: (1) fear, (2) anxiety, (3) sadness, (4) anger, and (5) hostility. The 5 items were averaged to create an additive index (mean 2.43, SD 0.81; Cronbach α=0.79).

#### Control Variables

Respondents’ demographic characteristics, such as age, gender, income, and education, were included as control variables. In addition, because risk perception, knowledge about COVID-19, and institutional trust tended to influence people’s negative emotions [54,55] and thereby affected trust gaps, they were also entered as covariates. Risk perception involves the judgment of the likelihood of being infected by the coronavirus and the assessment of the severity of an infection [56]. Knowledge is defined in two ways: (1) objective knowledge, which reflects the actual amount of information an individual has about a topic, and (2) subjective knowledge, which shows an individual’s self-evaluation of the acquired knowledge [57]. Given that respondents might overestimate their subjective knowledge of the coronavirus [58], this study focused on one’s objective knowledge about COVID-19. Institutional trust refers to people’s positive judgment of the trustworthiness of institutionalized organizations [59]. In public health emergencies, key institutional actors include government agencies, medical systems, and scientists. Thus, institutional trust in this study consists of people’s trust in governments, medical systems, and scientists.

Consistent with the established measurement of risk perception [53], risk perception of COVID-19 was calculated by multiplying perceived susceptibility by perceived severity (mean 7.22, SD 4.11). These two items were measured on a 5-point Likert scale: (1) perceived susceptibility: the likelihood of getting infected by COVID-19 between now and the near future (1=very unlikely, 5=very likely), and (2) perceived severity: the threat of COVID-19 infection to one’s health and life (1=not at all serious, 5=very serious). Referring to the measurement of MERS knowledge [38], knowledge about COVID-19 was measured by 5 quiz-type questions, such as “COVID-19 patients all have symptoms such as fever and cough” (wrong), and “patients who have underlying health conditions are more likely to suffer from severe COVID-19” (right). Correct answers were coded as 1, and incorrect answers were coded as 0 for each question. The answers to 5 questions were added to create the COVID-19 knowledge index (mean 4.57, SD 0.63).

Five-point Likert scales ranging from 1 (strongly disagree) to 5 (strongly agree) were used to measure 3 types of institutional trust. Based on the previous measurement [59], trust in government was measured by 6 items regarding the central and local governments, such as “What the central government has done so far in containing the coronavirus is trustworthy,” “I trust the local government’s capability to cope with the coronavirus.” Six items were averaged to create a composite index (mean 3.98, SD 0.66; Cronbach α=0.85). Five items were averaged to develop the index of trust in medical systems (mean 3.51, SD 0.75; Cronbach α=0.76). Example items included medical professionals “have high rates of correct diagnosis,” and “provide effective health care for patients” [60]. Trust in scientists was measured by respondents’ evaluation of scientists in terms of their trustworthiness, competence, benevolence, and general credibility [61] (mean 4.35, SD 0.56; Cronbach α=0.80).

### Statistical Analyses

We used PROCESS, an SPSS macro developed by Hayes [62], to test the hypotheses. One of the advantages of PROCESS is that it implements the recommended asymptotic and bootstrapping method to test hypotheses involving mediation effects that have few requirements for model assumption [63]. The research hypotheses constitute 2 mediation effects, in which negative emotions mediate the association between digital media use and the 2 trust gaps, respectively. Consequently, we chose model 4 in the PROCESS templates.

We separately ran 2 mediation tests. In the first mediation test, family members–strangers trust gap was entered as the dependent variable, digital media use as the independent variable, and negative emotions as the mediator variable. Age, gender, income, education, risk perception, COVID-19 knowledge, trust in government, trust in medical systems, and trust in scientists were entered as covariates. In the second mediation test, the family members–acquaintances trust gap was entered as the dependent variable, while other variables remained the same as the first test.

## Results

### Preliminary Analysis

Digital media use, negative emotions, and the control variables explained 2.5% of the variance in the family members–strangers trust gap (*F*_11,1556_=3.67; *P*<.001; *R*^2^=0.025) and 4.6% of the variance in the family members–acquaintances trust gap (*F*_11,1556_=6.86; *P*<.001; *R*^2^=0.046). Among the control variables, trust in scientists was positively associated with the family members–strangers trust gap (*B*=0.15, SE 0.05; *P*=.004), indicating that a higher level of trust in scientists during the pandemic tended to increase the gap between one’s trust in family members and in strangers. At the early stage of the outbreak, scientists advised people to practice social distancing to prevent the rapid spread of the coronavirus. By believing in scientists’ advice and frequently practicing social distancing, people’s trust in strangers decreased and thereby enlarged the family members–strangers trust gap. Besides, age (*B*=0.02, SE 0.003; *P*<.001), education (*B*=–0.07, SE 0.03; *P*=.01), and COVID-19 knowledge (*B*=–0.08, SE 0.04; *P*=.02) were significantly associated with the family members–acquaintances trust gap. In other words, older people tended to be more skeptical about acquaintances than their younger counterparts did. Besides, people who had greater education attainment and more COVID-19 knowledge might use a more rational approach to view the coronavirus than those with less education attainment or COVID-19 knowledge did, which lowered the former group’s distrust or skepticism in acquaintances.

### Testing Mediation Effects

After controlling for the effects of the control variables, digital media use was positively associated with negative emotions (*B*=0.17, SE 0.03; *P*<.001), and negative emotions were positively associated with the family members–strangers trust gap (*B*=0.15, SE 0.03; *P*<.001). According to the results of the bootstrap method at 95% CI, the indirect effect of digital media use on the family members–strangers trust gap via negative emotions was significant (*B*=0.03, SE 0.01; 95% CI 0.01-0.04), which supported hypothesis 1. However, the direct effect of digital media use on the family members–strangers trust gap was not significant (*B*=–0.03, SE 0.04, 95% CI –0.11 to 0.05).

In terms of the second hypothesis, digital media use was positively associated with negative emotions (*B*=0.17, SE 0.03; *P*<.001), while negative emotions were positively associated with the family members–acquaintances trust gap (*B*=0.08, SE 0.03; *P*=.01). The indirect effect of digital media use on the family members–acquaintances trust gap through negative emotions was significant (*B*=0.01, SE 0.01; 95% CI 0.003-0.027). Thus, hypothesis 2 was supported. Nevertheless, we did not observe a significant direct effect of digital media use on the family members–acquaintances trust gap (*B*=–0.04, SE 0.04; 95% CI –0.12 to 0.03).

**Figure 2:**
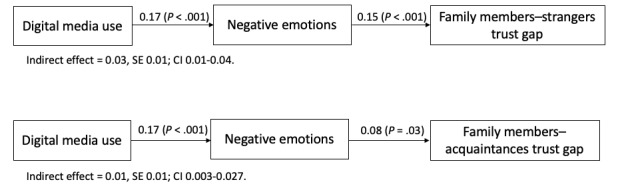
Diagrams of mediation effects. Unstandardized regression coefficients are reported.

These results revealed that negative emotions fully mediated the association between digital media use and the 2 trust gaps. Moreover, the mediation tests also demonstrated that the mediation effect of negative emotions on the family members–strangers trust gap was stronger than that on the family members–acquaintances trust gap, showing support for hypothesis 3. Tables 2 and 3 and Figure 2 demonstrate the statistical results and the final model, respectively.

**Table 2:** Regressing family members–strangers trust gap on antecedents^a^.

		Negative emotions		Family members–strangers trust gap	
		*B* (SE^b^)	*P* values	*B* (SE)	*P* values
**Control variables**					
	Age (years)	–0.003 (0.002)	–.07	0.004 (0.003)	.22
	Gender^c^	–0.14 (0.04)	<.001	0.02 (0.05)	.68
	Income	–0.003 (0.01)	.76	–0.004 (0.01)	.80
	Education	0.06 (0.02)	.009	–0.05 (0.03)	.09
	Risk perception	0.04 (0.005)	<.001	–0.01 (0.01)	.32
	COVID-19 knowledge	0.06 (0.03)	.05	–0.03 (0.04)	.40
	Trust in government	–0.20 (0.03)	<.001	0.07 (0.05)	.11
	Trust in medical systems	–0.18 (0.03)	<.001	0.02 (0.04)	.67
	Trust in scientists	0.04 (0.04)	.29	0.15 (0.05)	.003
**Antecedents**					
	Digital media use	0.17 (0.03)	<.001	–0.03 (0.04)	.51
	Negative emotions	—^d,e^	—	0.15 (0.03)^f,g^	<.001

^a^Unstandardized regression coefficients were reported.

^b^Standard errors are within parentheses; bootstrap sample size = 10,000.

^c^Gender: 0=female, 1=male.

^d^Not applicable.

^e^*R*^2^=16.6%; *F*_10,1557_=30.98; *P*<.001.

^f^*R*^2^=2.5%; *F*_11,1556_=3.67.

^g^Boot effects: *B*=0.03 (SE 0.01, 95% CI 0.01-0.04) for indirect effects; *B*=–0.03 (0.04, 95% CI –0.11 to 0.05) for direct effects.

**Table 3:** Regressing family members–acquaintances trust gap on antecedents^a^.

		Negative emotions		Family members–acquaintances trust gap	
		*B* (SE^b^)	*P* values	*B* (SE)	*P* values
**Control variables**					
	Age (years)	–0.003 (0.002)	.26	0.02 (0.003)	<.001
	Gender^c^	–0.14 (0.04)	<.001	0.08 (0.05)	.08
	Income	–0.003 (0.01)	.76	0.005 (0.01)	.72
	Education	0.06 (0.02)	.009	–0.07 (0.03)	.01
	Risk perception	0.04 (0.005)	<.001	–0.006 (0.01)	.29
	COVID-19 knowledge	0.06 (0.03)	.05	–0.08 (0.04)	.02
	Trust in government	–0.20 (0.03)	<.001	0.06 (0.04)	.15
	Trust in medical systems	–0.18 (0.03)	<.001	0.05 (0.04)	.18
	Trust in scientists	0.04 (0.04)	.29	–0.03 (0.05)	.56
**Antecedents**					
	Digital media use	0.17 (0.03)	<.001	–0.04 (0.04)	.29
	Negative emotions	—^d,e^	—	0.08 (0.03)^f,g^	.01

^a^Unstandardized regression coefficients were reported.

^b^Standard errors are within parentheses; bootstrap sample size = 10,000.

^c^Gender: 0=female, 1=male.

^d^Not applicable.

^e^*R*^2^=16.6%; *F*_10,1557_=30.98; *P*<.001.

^f^*R*^2^=4.6%, *F*_11,1556_=6.86.

^g^Boot effects: *B*=0.01 (SE 0.01, 95% CI 0.003-0.027) for indirect effects; *B*=–0.04 (0.04, 95% CI –0.12 to 0.03) for direct effects.

## Discussion

### Principal Findings

This study assessed the relationship between digital media use, negative emotions, the family members–strangers trust gap, and the family members–acquaintances trust gap during the COVID-19 pandemic in China. A majority of prior research used macrolevel perspectives to explain trust gaps. For instance, the differential mode of association (*chaxugeju*) emphasized that the kinship-based Chinese culture was the major cause of trust gaps, in which people trusted their family members more than others [11]. Besides, the rapid marketization and increasing mobility in China over the past few decades have enlarged the social distance between strangers and unfamiliar ones [64-66], thereby increasing trust gaps. Compared with this line of research, this study used a microlevel perspective and showed that individuals’ digital media use was an important predictor of trust gaps. Notably, the Chinese character *trust* (信) is composed of *people* (亻) and *words* (言), which indicates that what people say makes a difference to trust [67]. Moreover, due to the highly contagious nature of the coronavirus, people relied on digital media to acquire information and post comments during the pandemic. These findings indicate that what people said on digital media contributed to the change in their trust in others. Hence, in the digital society, digital media use serves as a valid approach to explicate trust gaps during public health emergencies.

The results demonstrated the mediating role of negative emotions in the associations between digital media use and 2 trust gaps. On the one hand, the positive association between digital media use and negative emotions was consistent with previous studies that focused on public health emergencies [19,20,38,39]. These studies showed that the wide use of digital media facilitated the spread of the virus-related information, which not only amplified people’s negative emotions but also resulted in emotion contagion among the population [68,69]. On the other hand, the positive associations between negative emotions and 2 trust gaps highlighted the role of negative emotions in motivating an individual to attribute the responsibility of a crisis to socially distant ones [41]. Negative emotions such as anxiety, anger, and hostility tend to create a sentiment of distrust in others, including strangers and acquaintances, thereby increasing the trust gaps. Prior research mainly focused on the impact of emotion contagion in social media on individual behaviors during public health crises [69,70]. In comparison, by examining the impact of negative emotions on 2 trust gaps, our study adds some insights into understanding how emotion contagion influences interpersonal ties in the digital environment.

By comparing the indirect effect of digital media use on 2 trust gaps via negative emotions, we found that negative emotions tended to enlarge the family members–strangers trust gap to a larger extent than the family members–acquaintances trust gap. These findings not only echo that strong aversive emotions result in individuals’ distrust in strangers or loosely connected ones [43-45] but also indicate that people tend to attribute more responsibility to socially distant others, thereby leading to more distrust in strangers than in acquaintances. In recent years, strangers have been increasingly distanced as untrustworthy others, which suggests a decrease in social trust [66,71]. These findings remind us to stay alert to the widening family members–strangers trust gap caused by frequent digital media use and associated negative emotions.

In the past few years, the COVID-19 pandemic has exerted a profound influence on the ways people think, behave, and interact with others. Although the World Health Organization ended global health emergency declaration for the COVID-19 pandemic on May 5, 2023 [1], large-scale infectious diseases might break out in the future [5]. Moreover, the postpandemic era is increasingly shaped by the use of various digital tools [72]. As long as biased and uncivil comments about out-group members circulate on digital media, people’s exposure to this information tends to amplify their negative emotions, which in turn enlarges 2 trust gaps. Thus, the findings of this study have implications for curbing the enlargement of trust gaps in the postpandemic era.

We suggest policy makers to take measures to pursue an emotionally balanced digital media environment with less uncivil, hostile, or sensational messages about out-group members when an infectious disease breaks out. For instance, the emotionally laden web-based misinformation and disinformation should be identified and labeled to remind users to keep alert of these contents. Besides, algorithms that push diverse viewpoints to users can be used to avoid emotional polarization. These efforts may ease people’s negative emotions during their digital media use, thereby curbing the enlargement of trust gaps. Meanwhile, we advise digital media users to improve their verification skills. When encountering a piece of news on digital media, users should first verify the credentials of the authors, such as whether the authors are specialized in the field covered by the news. Then, users should check the source by asking these questions: Are there any cited sources in the article? How many sources are cited? How credible are the sources? Finally, users should examine whether the news article includes biased or sensational contents. The 3-step verification behavior helps users avoid the overamplification of negative emotions and prevent the enlargement of trust gaps, even if they encounter fake news on digital media.

### Limitations and Future Directions

This study has several limitations. First, the measurement of digital media use might be too general to capture its precise impact on negative emotions and trust gaps. Although frequency of exposure to information may provide much information about the influence of digital media use on users’ perceptions [73], researchers could analyze the content related to negative emotions and distrust in others on digital media to replicate this study. In addition, although prior studies have demonstrated that people primarily experience negative emotions at the early stage of a public health emergency [35,38,39], they may also feel positive emotions, such as hope, to navigate them through uncertainties [74,75]. Accordingly, future research could incorporate positive emotions into the model to get a more nuanced and comprehensive understanding of how digital media use influences social trust–related outcomes through negative and positive emotions. Besides, although the single-item measurement of trust in certain groups has been frequently used [76,77], multiple items should be used to measure one’s trust in family members, acquaintances, and strangers in the future to improve the construct reliability.

Second, in terms of data collection, the cross-sectional web-based survey provided empirical evidence for testing the associations between the examined variables. However, we cannot claim causality in the proposed model. It is possible that participants who experienced negative emotions and distrusted strangers and acquaintances selected to expose themselves to biased and uncivil contents to confirm their existing perceptions. Moreover, given that negative emotions were highly volatile over the course of the pandemic [78], researchers are advised to use multiple waves of survey that focus on different stages of a public health crisis in the future. This longitudinal design not only enables a researcher to trace the evolvement of negative emotions but also warrants the examination of the causal relationships between digital media use, negative emotions, and trust gaps.

Finally, our survey was conducted at the onset of the pandemic. The early stage of a pandemic was characterized by high public demand for information [79], strong negative emotions [80], and a fragile state of social trust [81]. Conducting the survey at the middle or late stages of the pandemic might have yielded different results. Therefore, the findings should be interpreted as specific to the initial stage of a public health emergency, instead of being generalized to other phases. However, as an exploratory study, the current model may serve as a link between the early-stage trust dynamics and their long-term impacts in the postpandemic era. Researchers can use our findings to explicate trust-related outcomes in the present days.

### Conclusions

The widening trust gaps amid public health emergencies not only undermine social integration but also hinder postpandemic recovery. This study examines trust gaps in the early phase of the COVID-19 pandemic in China and demonstrates that frequent digital media use was positively associated with negative emotions. These emotions, in turn, were positively associated with 2 trust gaps: the family members–strangers trust gap and the family members–acquaintances trust gap. Moreover, the mediation effect of negative emotions between digital media use and the family members–strangers trust gap was stronger than that between digital media use and the family members–acquaintances trust gap. Compared with previous macro-level studies that focused on factors such as culture and modernity to explain the decline in social trust [11,82], our study contributes in two ways: (1) delineating the structure of decreasing social trust into 2 distinct trust gaps, and (2) using a media effect approach to unveiling the individual-level mechanism that contributes to the widening trust gaps. Against the backdrop of the mediatized era and global risk society, the findings underscore the importance of the appropriate use of digital media and the strategic management of public emotions to curb the widening trust gaps. This, in turn, can facilitate effective public health crisis intervention in the postpandemic era.

## Abbreviations

CNY: Chinese Yuan
MERS: Middle East respiratory syndrome
